# Use of intraoperative local field potential spectral analysis to differentiate basal ganglia structures in Parkinson's disease patients

**DOI:** 10.14814/phy2.13322

**Published:** 2017-06-22

**Authors:** Rachel Kolb, Aviva Abosch, Gidon Felsen, John A. Thompson

**Affiliations:** ^1^ Department of BioengineeringUniversity of Colorado School of MedicineAuroraColoradoUSA; ^2^ Department of NeurosurgeryUniversity of Colorado School of MedicineAuroraColoradoUSA; ^3^ Department of Physiology and BiophysicsUniversity of Colorado School of MedicineAuroraColoradoUSA

**Keywords:** Basal ganglia, deep brain stimulation, local field potential, Parkinson's disease

## Abstract

Identification of brain structures traversed during implantation of deep brain‐stimulating (DBS) electrodes into the subthalamic nucleus (STN‐DBS) for the treatment of Parkinson's disease (PD) frequently relies on subjective correspondence between kinesthetic response and multiunit activity. However, recent work suggests that local field potentials (LFP) could be used as a more robust signal to objectively differentiate subcortical structures. The goal of this study was to analyze the spectral properties of LFP collected during STN‐DBS in order to objectively identify commonly traversed brain regions and improve our understanding of aberrant oscillations in the PD‐related pathophysiological cortico‐basal ganglia network. In 21 PD patients, LFP were collected and analyzed during STN‐DBS implantation surgery. Spectral power for delta‐, theta‐, alpha‐, low‐beta‐, and high‐beta‐frequency bands was assessed at multiple depths throughout the subcortical structures traversed on the trajectory to the ventral border of STN. Similar to previous findings, beta‐band oscillations had an increased magnitude within the borders of the motor‐related area of STN, however, across several subjects, we also observed increased high‐beta magnitude within the borders of thalamus. Comparing across all patients using relative power, we observed a gradual increase in the magnitude of both low‐ and high‐beta‐frequency bands as the electrode descended from striatum to STN. These results were also compared with frequency bands below beta, and similar trends were observed. Our results suggest that LFP signals recorded during the implantation of a DBS electrode evince distinct oscillatory signatures that distinguish subcortical structures.

## Introduction

Parkinson's disease (PD), a movement disorder characterized by bradykinesia, rigidity, and tremor, results from progressive loss of nigrostriatal dopaminergic innervation (Obeso et al. 2000). Neuroanatomical and electrophysiological studies in animal models of parkinsonism that mimic neuronal loss in the substantia nigra pars compacta (SNc) reveal widespread dysregulation throughout the basal ganglia (BG) circuitry (Ungerstedt 1968; Burns et al. 1983; Waters et al. 1987; Smeyne and Jackson‐Lewis 2005). In human PD, post mortem analysis confirms that dopaminergic degeneration affects all levels of the motor system mediating selection and execution of motor output (Braak et al. 2003).

The BG is composed of several complex parallel loops that integrate distinct cortical areas (Alexander et al. 1986; Wichmann and DeLong 1998; Wichmann et al. 2000), and the classical model of BG function in PD, predicated on dopamine deficiency, has proven successful in identifying critical therapeutic targets – initially for ablation, and more recently for deep brain stimulation (DBS) (Ghika et al. 1998; Krack et al. 1998; Kumar et al. 1998; Limousin et al. 1998). In the context of DBS surgery, electrophysiological recordings for intraoperative anatomical mapping or from the DBS electrode contacts have led to the development of new models of PD neuropathophysiology. These models have been developed from the study of oscillatory patterns in the cortico‐basal‐ganglia (CBG) pathway (Brown et al. 2001; Priori et al. 2004; Weinberger et al. 2006).

Studies in nonhuman primates have linked aberrant oscillations in several regions throughout the CBG with PD motor impairments (Bergman et al. 1994; Nini et al. 1995). These pathological oscillations in the CBG exhibit increased synchrony and increased amplitude (Bezard et al. 2001). Similarly, neurophysiological observations in PD patients implanted with DBS electrodes have revealed the existence of abnormal oscillatory activity in local field potentials (LFP) recorded from globus pallidus internal segment (GPi) and subthalamic nucleus (STN), the therapeutic targets of stimulation (Brown et al. 2001; Brown 2003).

High‐amplitude beta‐band oscillations, which have garnered the greatest interest in the LFP spectrum for their putative role in PD pathophysiology, are detected predominantly in the motor territory of STN, respond to dopamine replacement, and correlate with bradykinesia and rigidity (Brown et al. 2001; Amirnovin et al. 2004; Zaidel et al. 2009, 2010; Lopez‐Azcarate et al. 2010). In human PD subjects, aberrant beta‐band signals have been observed throughout the CBG, including STN, GPi, and substantia nigra pars reticulata (SNr) (Weinberger et al. 2006; Alavi et al. 2013). Despite extensive exploration of beta‐band oscillations in the PD‐related pathophysiological CBG network, questions persist regarding the onset and origin of aberrant beta activity within the CBG (Levy et al. 2000, 2001; Moran et al. 2008), and their utility as a biomarker to inform closed‐loop DBS therapies (Telkes et al. 2014; Thompson et al. 2014).

In this study, we examined LFP, which reflect the summed response of synaptic potentials that encompass a 1 mm^2^ volume of tissue around the electrode (Buzsáki et al. 2012). Here, we sought to examine the intra‐ and intersubject similarity and variability in spectral properties of LFP throughout the CBG circuit traversed during the standard DBS implant surgery. Importantly, we describe the oscillatory signatures within and between different brain structures of the CBG to assess whether changes in the modulation of specific frequency bands at the transitions between structures or within structures inform pathophysiological circuit dynamics. In addition, LFP signals provide a more robust electrophysiological readout as compared to the traditional single‐neuron approach of microelectrode recording, and have been successfully employed in computational models to assist in DBS target optimization (Zaidel et al. 2010; Telkes et al. 2014, 2016).

## Methods

This investigation was approved by the Colorado Multiple Institution Review Board (COMIRB). All LFP analyses were conducted on de‐identified data collected during the standard‐of‐care procedures for routine STN‐DBS implantation surgery in patients with idiopathic PD, using a retrospective chart review IRB‐approved protocol. Study population consisted of 21 PD patients (10 males, average age 62.63 ± 7.1 years).

The operative procedure for DBS implantation surgery has been described in detail previously (Abosch et al. 2013). Subsequent to standard stereotactic imaging methods for trajectory and target planning of DBS electrodes (Abosch et al. 2002; Telkes et al. 2016), intraoperative microelectrode recordings were used to optimize STN localization. LFP signals were recorded from the semimacroelectrode (1 mm wide, 3 mm proximal to the tip of the microelectrode) of three electrodes (NeuroProbe, AlphaOmega Inc., Nazareth). Electrodes were positioned using a standard microelectrode holder that permits parallel recordings from up to five electrodes. One electrode (designated “center”) was always positioned in register with the imaging‐derived target in posterior‐ventral STN, with a trajectory that began at the coronal suture, avoided the ventricle, and was 55–60° from the plane connecting the anterior and posterior commissure. The remaining two positions were selected based on individual STN anatomy (relative to “center”: anterior 38%, posterior 2%, medial 30%, lateral 26%; percent of total number of trajectories excluding the center track). Signals were collected at 1.3 kHz, and referenced to the electrode cannula. A NeuroDrive (AlphaOmega Inc., Alpharette, GA) microdrive was used to advance the electrodes simultaneously into the brain, at submillimetric steps, and transmit data to the recording system (MicroGuide or NeuroOmega; AlphaOmega Inc., USA).

Electrophysiological recordings began at 25 mm above target, with target defined as the ventral‐posterior border of STN, and advanced in steps of 100–1000 mm. At each step, a recording sample (between 10 and 430 sec; 43 ± 58 sec) was collected, along with a digital stamp of the depth with respect to the stereotactic target, in micrometer resolution. For each depth, a neurosurgeon and neurophysiologist, well‐experienced with intraoperative electrophysiological assessment, determined the approximate location of the recording within the brain, based on auditory and visual analysis of single‐neuron activity profile. The standard trajectory used for targeting STN traversed striatum, thalamus, and STN. Intraoperative assessment of brain localization was recorded and used to annotate recording depths. All subsequent analyses were computed offline. Data used in this analysis were derived from 81 electrode trajectories, recorded during 27 DBS implant surgeries, from 21 unique patients. Within each trajectory, only traces recorded for longer than 10 sec were stored for further postprocessing and analysis.

### Preprocessing raw LFP signals

**Figure 1:**
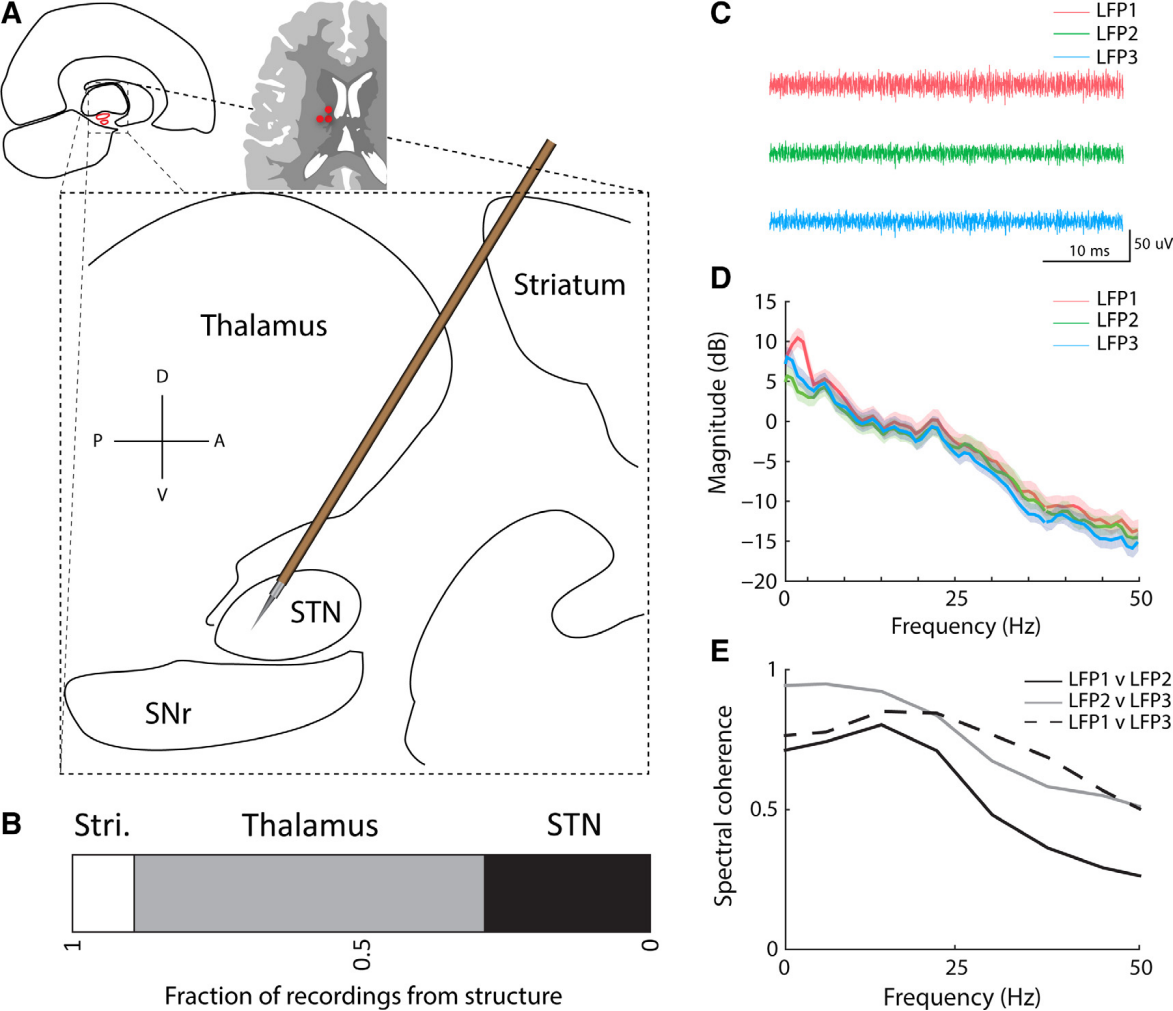
A sagittal representation of local field potential recording locations and representative traces. (A) Sagittal flat map depicting deep brain structures traversed during routine implantation of a deep brain stimulation electrode into the subthalamic nucleus (STN). D = dorsal; A = anterior; V = ventral; P = posterior. Top left inset depicts an axial section schematic of the brain highlighting the most common electrode configuration used for microelectrode recording (three red dots): three electrodes in the orientation center, anterior, and lateral. (B) Fraction of individual recordings that were derived for each of the three brain structures under investigation—striatum (Stri.), thalamus, and STN. The number in each box represents the number of recordings from the specified structure. (C) Three representative raw local field potential recordings simultaneously acquired during an STN‐DBS implantation surgery from a three‐electrode array separated by a distance of 2 mm (LFP 1 = center, 2 = medial, 3 = anterior). LFP signals were recorded at 4.3 mm from target (ventral border of STN) and designated within STN based on interpretation of microelectrode recordings. (D) The power spectral density (PSD) of the three representative local field potential recordings shown in (C). (E) The spectral coherence computed between the pairwise comparisons of the PSD shown in (C).

All recorded data were imported into MATLAB (Mathworks; Natick, Massachusetts). Raw signals were visualized within the time domain, and individual traces with clear recording artifacts were excluded. To confirm that the time domain signals collected for each LFP electrode were unique and distinct, we computed the magnitude‐squared signal coherence for each pair of electrodes, at each depth (i.e., LFP1 vs. LFP2, LFP1 vs. LFP3, and LFP2 vs.LFP3; see Fig. 1E). The coherence was calculated using Welch's modified periodogram method with a hamming window of 100, overlap of 80 samples, and an FFT length of 100 points. Each LFP signal was compared to the other recorded signals, and the resulting one‐sided coherence estimate indicated that each signal was indeed distinct. Each LFP recording was processed using the following steps: (1) detrended by subtracting the mean, and (2) a bandpass filter was designed as a fourth‐order low‐pass zero‐phase lag infinite impulse response (IIR) filter, with a cutoff frequency of 350 Hz. These filter attributes were chosen because the processing was completed offline, and the entire dataset was available for filtering. The cutoff frequency was determined using the Nyquist criterion that the sampling frequency should be at least twice the bandwidth of the highest frequency being analyzed. To remove remaining signal artifacts, additional processing steps were carried out in order to prepare the data for analysis. Depth recordings with values greater than six times the standard deviation above the mean, likely caused by noise, were eliminated from analyses.

### Signal analysis

Analysis of the signals within the frequency domain began by calculating the power spectral density in decibels. Welch's method was used to estimate the power of a signal at different frequencies using periodogram spectrum estimates to reduce noise. To calculate the power spectrum, a window of 650 samples was used, with an overlap of 50 samples. This was repeated for each patient, across all depths, and for electrodes individually. LFP signals were aggregated by brain structure based on the expert interpretation of the multiunit MER activity by an experienced neurophysiologist. MER signals were categorized separately for each electrode, as entry and exit into a brain structure (e.g., the thalamus) by concurrently driven electrodes, offset by 2 mm, are not expected to occur at the same depths. In order to compare across multiple patients, the relative power was calculated. Relative power was normalized on a per‐patient and per‐electrode basis, using the average power for each frequency bin collected from samples identified in the striatum. All signals within each structure from a given electrode were then normalized to that value to compare signals across patients. We noted that outliers remained in the data when looking at relative power using this calculation method, so any resulting values outside of 3 standard deviations from the mean were defined as outliers, and removed prior to any statistical testing.

### Statistical analysis

All statistical analyses were computed using Matlab 2016b (Machine Learning and Statistics Toolbox; Mathworks; Natick, MA). In order to compare across all three brain structures—striatum, thalamus, and STN— for multiple patients, a one‐way analysis of variance (ANOVA) or nonparametric ANOVA (Kruskal–Wallis H test) was computed on group (brain structure) means for relative power data. Post hoc (Tukey's Honest Significance Difference Criterion or Dunn's test) comparisons were used to compare between structures for different frequency bands lower (13–20 Hz) and higher (21–30 Hz) beta frequencies along with the frequency bands below beta: delta (<3 Hz), theta (4–7 Hz), and alpha (8–12).

## Results

We analyzed LFP data from 1454 intraoperative isolated recordings, acquired from 81 electrode tracks, in 27 STN‐DBS surgeries (14 Left and 13 Right STN implantations), conducted in 21 PD patients. For each surgery, three parallel electrode trajectories, spaced 2 mm apart, afforded recording epochs at multiple depths (36.6 ± 12.3; mean ± STD) per‐electrode track. A schematic of the common trajectory for STN‐DBS implantation is provided in Figure 1A, highlighting the four structures most frequently traversed: striatum, thalamus, STN, and SNr. The horizontal stacked bar chart (Fig. 1B) represents the proportion of experimental recordings that was derived from each of the three structures under study in this report: striatum, thalamus, and STN. Figure 1 depicts representative unfiltered LFP traces (1C), power spectral density (PSD; 1D), and magnitude‐squared coherence (1E) for three parallel electrode recordings obtained at a depth of 5 mm dorsal to the ventral border of STN. Coherence and PSD both demonstrate that the parallel recordings detect distinct electrical fields, despite the proximity of recording sites (see Methods for description of recording apparatus).

### Differences in beta‐frequency oscillations across BG structures

**Figure 2:**
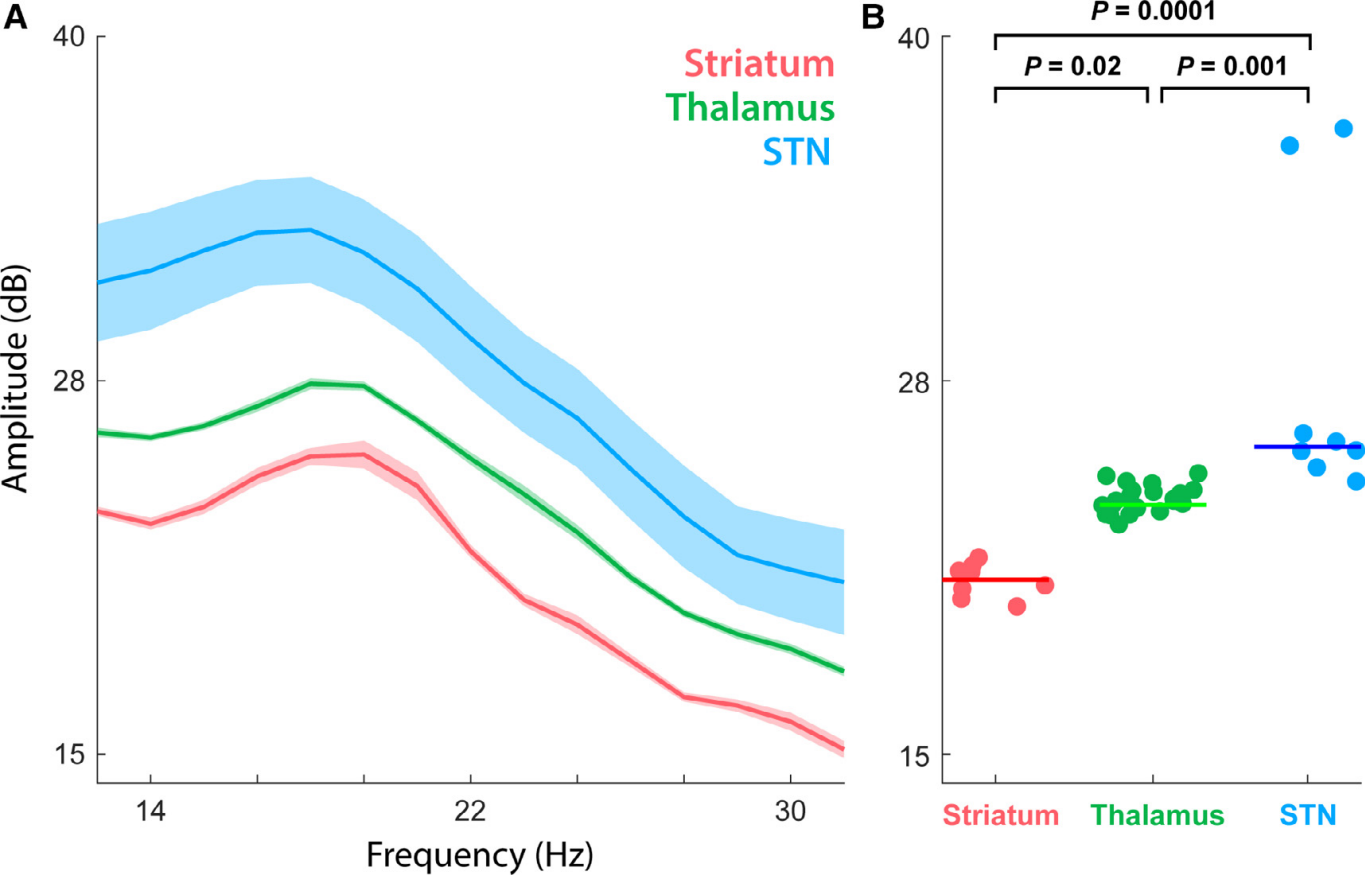
Representative amplitude differences in the beta band for striatum (Stri), thalamus (Thal), and subthalamic nucleus (STN). (A) The power spectral density (PSD) estimate in the beta band (13–30 Hz) for the Stri, Thal, and STN. Ribbon plot depicts the mean and SEM (standard error of mean) for all recordings that occurred within each structure averaged for all three electrodes. (B) Statistical analysis (Kruskal–Wallis H test) of the individual patient samples summarized in (A) determined that the structures were significantly separable based on beta‐band power (*χ* ^2^(2) = 25.26, *P *=* *3.2 × 10^−6^ striatum vs. thalamus, *P *=* *0.02; striatum vs. STN,* P *<* *0.0001; thalamus vs. STN,* P *=* *0.001). The mean rank of the signal across the trajectory through STN is higher than the signal mean for thalamus or striatum, with a mean striatum signal of 4.5 dB, thalamus of 21.12 dB, and STN of 37.13 dB (mean ± STD).

Previous reports in the literature have documented significant increases in LFP oscillatory activity and power magnitude within STN for the beta‐frequency band, recorded in patients undergoing DBS implantation (Cassidy et al. 2002; Kühn et al. 2004; Alegre et al. 2005; Chen et al. 2006; Gatev et al. 2006; Weinberger et al. 2006; Thompson et al. 2014). In this study, we sought to compare different spectral properties of the LFP signal, with respect to the unique brain structures traversed during STN‐DBS implantation, beginning with the LFP beta band (13–30 Hz; (Brown and Williams 2005; Fogelson et al. 2006; Rossi et al. 2008; de Hemptinne et al. 2015)). Figure 2A–B represents the mean beta‐band (± SEM) spectral power for striatum, thalamus, and STN, for a single surgery confirmed along the trajectory. Comparing the individual epoch recordings that occurred within each structure demonstrates that the beta band is sufficient to distinguish between these three brain areas in this subject (Kruskal–Wallis *H* test, *χ* ^2^(2) = 25.26, *P *=* *3.2 × 10^−6^ striatum vs. thalamus, *P *=* *0.02; striatum vs. STN, *P *<* *0.0001; thalamus vs. STN, *P *=* *0.001). Beta‐frequency band power is noted to increase as the recording depth descends from striatum to thalamus, and from thalamus to STN, across all three electrodes. The mean rank of the signal across the trajectory through STN is higher than the signal mean for thalamus or striatum, with a mean striatal signal of 4.5 dB, thalamic of 21.12 dB, and STN of 37.13 dB (mean ± STD).

**Figure 3:**
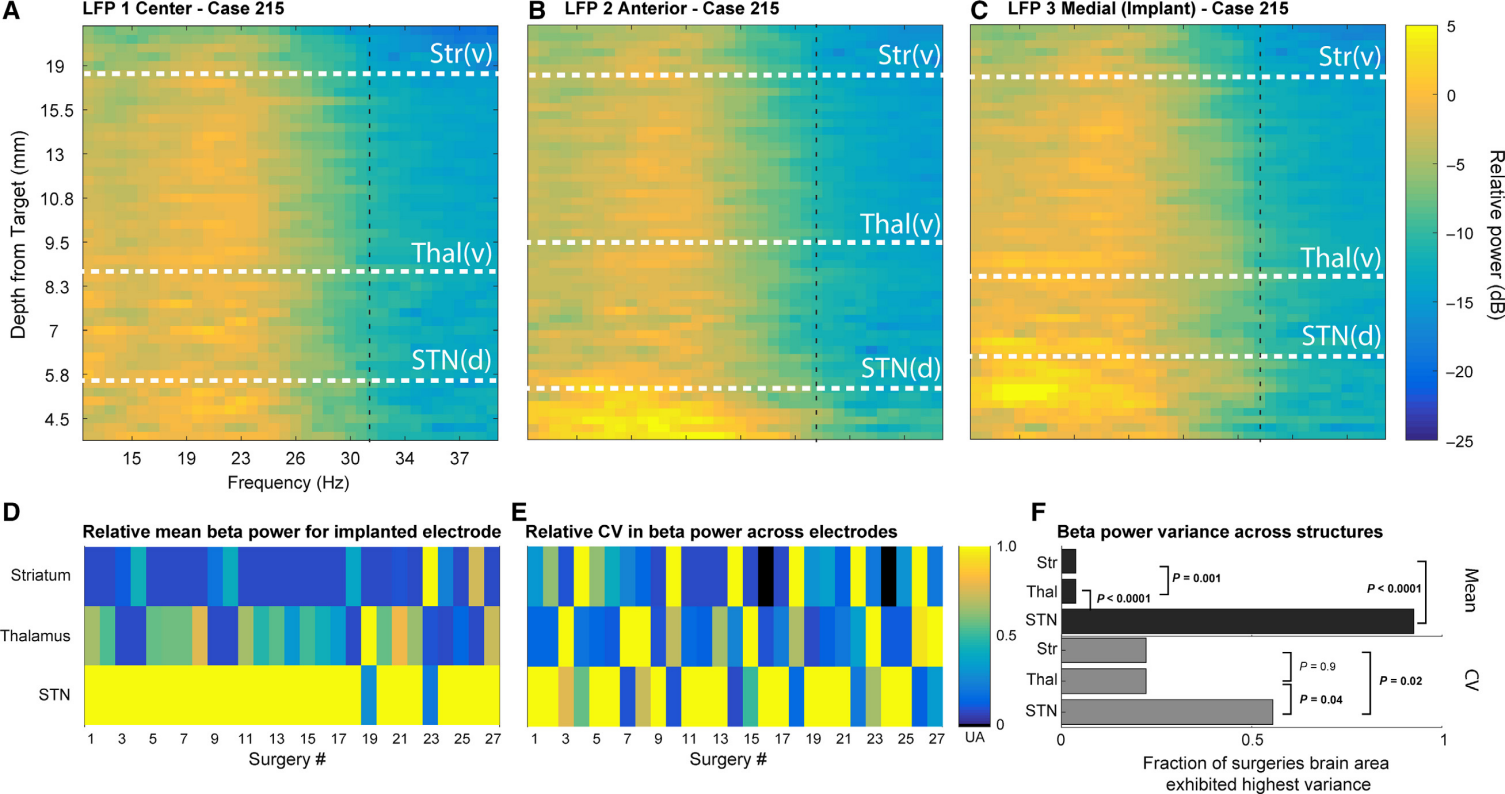
Spectrogram of recording depth over frequency for the beta band. (A–C) Spectrogram of depth over frequency for the beta band (12–32 Hz) acquired from three simultaneously recorded electrodes during a representative STN deep brain‐stimulating (DBS) surgery. Each graph represents an electrode from a different Ben‐Gun position: (A) center (derived from direct targeting on preoperative stereotactic imaging), (B) 2 mm anterior to the center track, and (C) 2 mm medial to the center track; trajectory selected for DBS implantation based on single‐unit recordings and intraoperative stimulation mapping for benefits and side effects. White horizontal lines indicate the borders of the specific brain structures along the trajectory identified using single unit recordings, vertical black lines indicate borders of beta‐band frequencies. (D) Normalized mean beta power across structures traversed within the electrode trajectory selected for DBS implantation. Mean beta power for each structure was normalized by the structure exhibited the highest mean power. Each column represents the electrode trajectory selected for DBS implantation for each of 27 surgeries. *E,* The coefficient of variation for each structure across all three electrodes for each of the 27 surgeries. *F*, Top horizontal bar chart (black bars) summarizes relative mean beta power across implanted electrodes. Statistical analysis using one‐way ANOVA for structure shows that relative mean beta is highest in STN and the thalamus mean beta is significantly higher than striatum (*P* < 0.01 for all three comparisons; group means for STN, thalamus, and striatum, respectively: 0.93, 0.37, and 0.11). Bottom horizontal bar chart (gray bars) summarizes CV analysis across all surgeries. For the beta band, across surgeries, STN exhibited the highest CV compared to both thalamus (*P *<* *0.04) and striatum (*P *<* *0.02) and striatum was not statistically different from thalamus (*P* = 0.9).

In order to confirm that the findings were not specific only to certain depths along the trajectory, we computed the PSD for all recordings confirmed within the striatum, thalamus, and STN, for each individual electrode trajectory. Figure 3A–C depicts all three parallel electrode recordings from a single STN‐DBS implantation surgery (LFP‐1 trajectory derived from direct targeting off of stereotactic imaging, LFP‐2 was 2 mm anterior of LFP 1, and LFP‐3 was 2 mm medial of LFP 1; LFP‐2 was the trajectory selected for DBS implantation). For Figure 3A–C, the white dotted lines demarcate structure boundaries: ventral border of striatum, ventral border of thalamus, and dorsal border of STN. The pseudocolored heat plots highlight the increase in beta‐frequency magnitude at the recording depths associated with STN (i.e., LFP 2 and 3). To assess whether increased beta power in STN typified the implanted trajectory across surgeries, we examined the relative power averaged for each structure within the electrode trajectory selected for implantation for all surgeries (Fig. 3D). We observed that in the majority of surgeries, STN evinced the highest beta power across structures, within the implanted track. In a second population analysis, we sought to estimate the relative variability in beta power across structures for all subjects. In particular, dorsal and ventral STN have been differentiated by their relative beta power modulation (Zaidel et al. 2010). To address this question in our data, we computed the relative coefficient of variation (averaged across all three electrodes) for each structure, for all surgeries. Figure 3E–F indicate that across subjects, STN shows the highest relative variability in beta power in comparison to thalamus, but not striatum, and no differences in variability are observed between thalamus and striatum (Kruskal–Wallis *H* test, *χ* ^2^(2) = 7.4, *P *=* *0.02 striatum vs. thalamus, *P *=* *0.99; striatum vs. STN, *P *=* *0.051; thalamus vs. STN, *P *=* *0.047). In Figure 3, our findings within STN are consistent with prior reports that describe an increase in LFP power within the borders of STN (Kühn et al. 2005; Weinberger et al. 2006; Zaidel et al. 2010; Telkes et al. 2014). Across STN‐DBS surgeries, it is evident that within the beta band, there is an observable increase in PSD as the electrode enters STN, specifically focused within the lower frequencies of the beta band. The intensity and width of the increase in PSD varies for patients, but is clearly located near the dorsal border of STN.

### Average relative beta power continuously increased from striatum to STN

**Figure 4:**
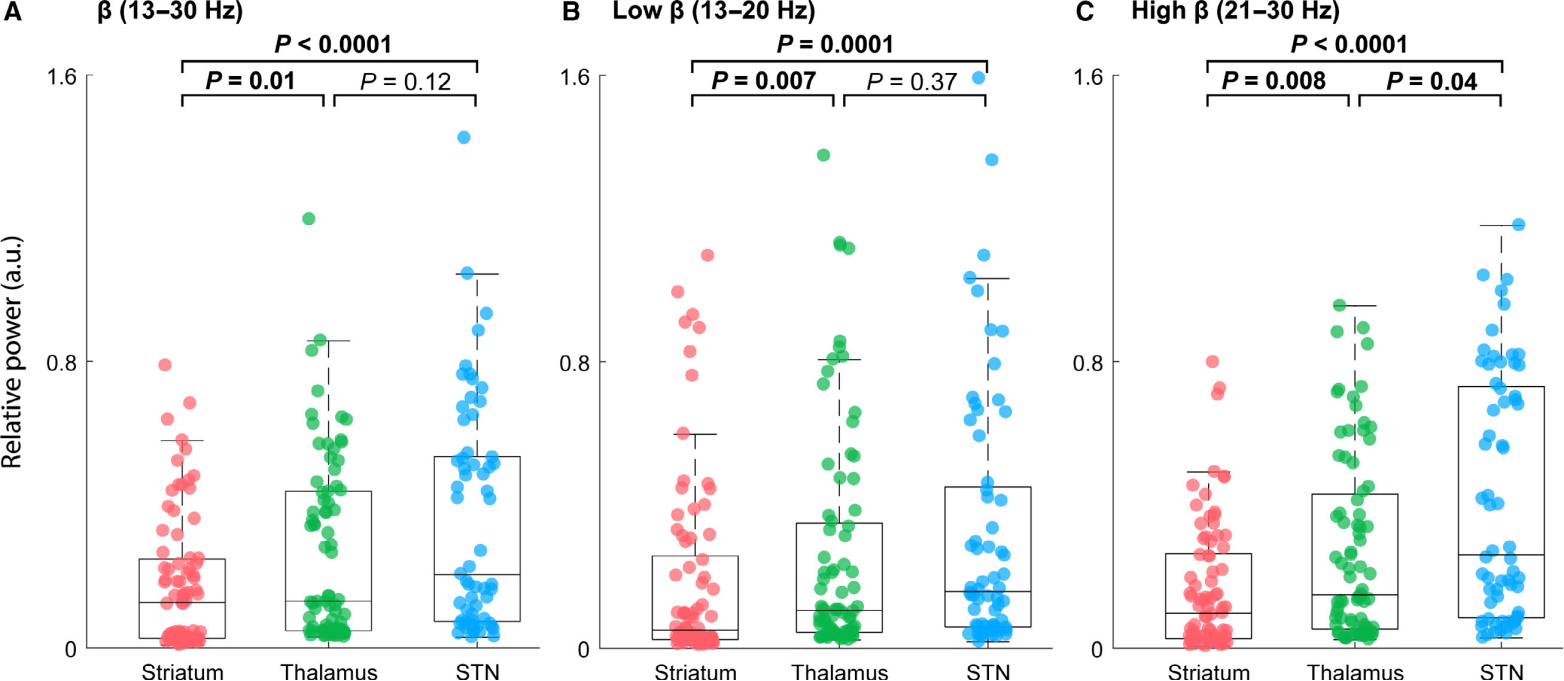
Relative power across structures for all subjects within the beta band. (A) Boxplot of the relative power spectral density for the entire beta‐frequency spectrum (13–30 Hz) comparing striatum, thalamus, and STN for all subjects. Both STN and thalamus exhibited higher‐beta‐band power than striatum. *P* values: striatum versus thalamus, *P *=* *0.01; striatum versus STN,* P *<* *0.0001; thalamus versus STN,* P *=* *0.12. (B) Similar representation to (A) with respect to low beta frequencies (13–20 Hz). Both STN and thalamus exhibited higher‐beta‐band power than striatum. *P* values: striatum versus thalamus, *P *=* *0.007; striatum versus STN,* P *=* *0.0001; thalamus versus STN,* P *=* *0.37. (C) Similar representation to (A) with respect to high‐beta frequencies (21–30 Hz). STN exhibited higher high‐beta‐band power than both thalamus and striatum and thalamus exhibited higher high‐beta power than striatum. *P* values: striatum versus thalamus, *P *=* *0.008; striatum versus STN,* P *<* *0.0001; thalamus versus STN,* P *=* *0.04.

We next sought to assess whether the discrete brain areas traversed during STN‐DBS implantation surgery would differ in their expression of beta‐band power across patients. Focusing on each specific structure, we compared the average relative power for total (13–30 Hz), low‐ (13–20 Hz), and high‐ (21–30 Hz) beta frequency in the striatum, thalamus, and STN, for all patients (Fig. 4A–C). To compare the relative band power across structures, we first normalized power for each patient, based on the average band‐specific magnitude in striatum, for each electrode. Comparison across brain structures for the total beta band revealed that both STN and thalamus exhibited higher‐beta‐band power than striatum (Kruskal–Wallis *H* test, *χ* ^2^(2) = 23.04, *P *=* *9.91 × 10^−6^; striatum vs. thalamus, *P *=* *0.01; striatum vs. STN, *P *<* *0.0001; thalamus vs. STN, *P *=* *0.12; mean ranks: 87.35, 117.05, 138.70, striatum, thalamus, STN, respectively). In Figure 4B–C, we further parsed the beta band into low‐ and high‐frequency bands based on observations that the lower beta frequencies are more likely to show greater power modulation in STN. No change in the relationship between the three structures was observed for low beta (low beta: Kruskal–Wallis *H* test, *χ* ^2^(2) = 19.35, *P *=* *6.27 × 10^−5^; striatum vs. thalamus, *P *=* *0.007; striatum vs. STN, *P *=* *0.0001; thalamus vs. STN, *P *=* *0.37) (mean ranks: 88.40, 119.56, 134.26, striatum, thalamus, STN, respectively). However, for high beta we found that across all patients all three structures were differentiable from each other. STN had the highest expression of high beta, followed by thalamus and, finally, striatum exhibited the lowest high‐beta power (high beta: Kruskal–Wallis *H* test, *χ* ^2^(2) = 28.68, *P *=* *5.91 × 10^−7^; striatum vs. thalamus, *P *=* *0.008; striatum vs. STN, *P *<* *0.0001; thalamus vs. STN, *P *=* *0.04) (mean ranks: 82.16, 115.94, 142.86, striatum, thalamus, STN, respectively).

### Differences in lower‐frequency oscillations across BG structures

**Figure 5:**
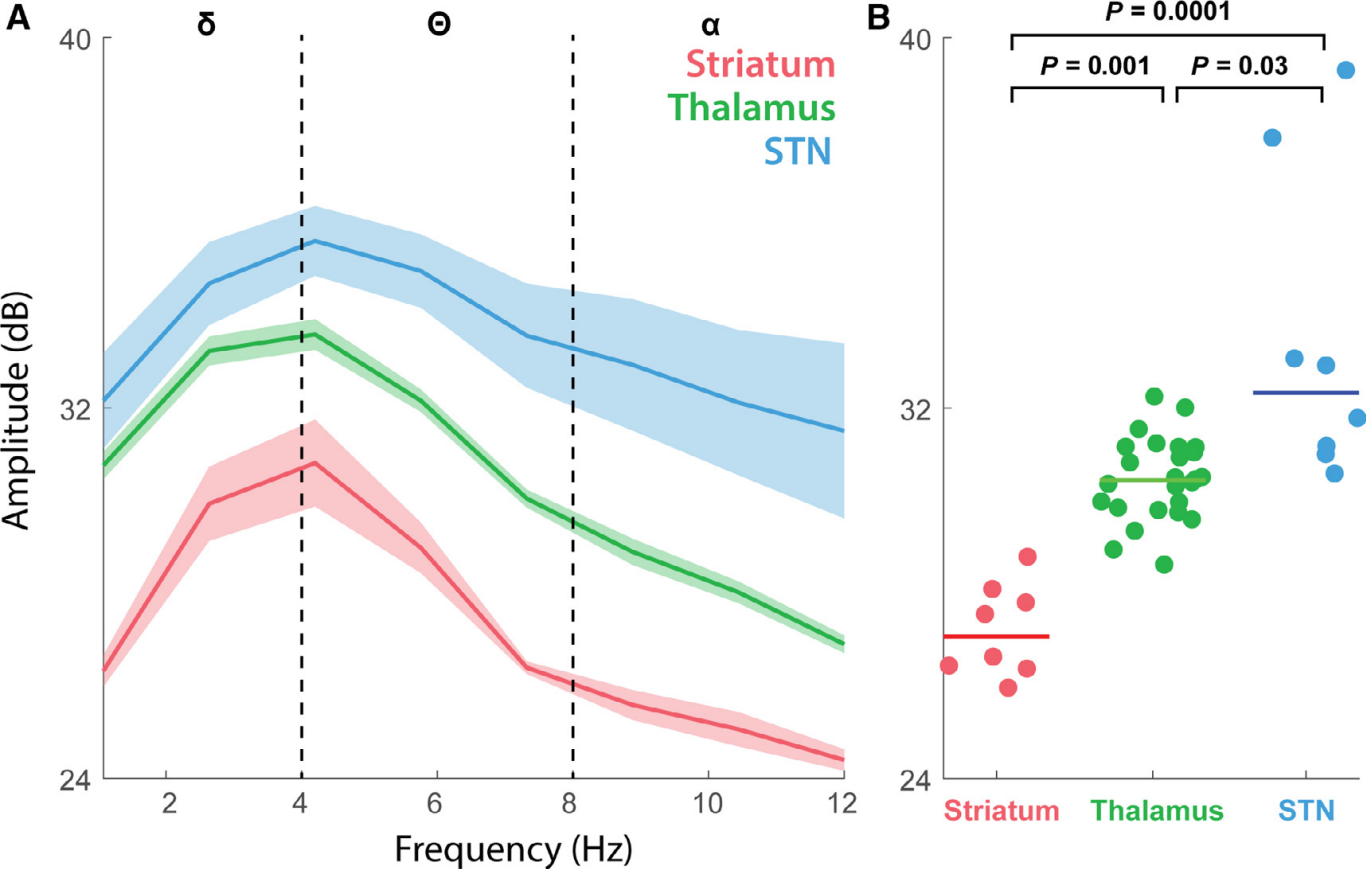
Representative amplitude differences in the *δ*,* θ,* and *α* bands for distinct deep brain structures. (A) The power spectral density (PSD) estimate in the delta band (1–4 Hz), theta (5–8 Hz), and alpha (9–11 Hz) frequency bands for the striatum, thalamus, and STN. Ribbon plot depicts the mean and SEM (standard error of mean) for all recordings that occurred within each structure averaged for all three electrodes. (B) Statistical analysis (Kruskal–Wallis H test) of the individual patient samples summarized in (A) determined that the structures were significantly separable based on the aggregate of the lower band power (*χ* ^2^(2) = 25.26, *P *=* *3.2 × 10^−6^ striatum vs. thalamus, *P *=* *0.001; striatum vs. STN,* P *<* *0.0001; thalamus vs. STN,* P *=* *0.03). The mean rank of the signal across the trajectory through STN is higher than the signal mean for thalamus or striatum, with a mean striatum signal of 4.63 dB, thalamus of 21.92 dB, and STN of 34.50 dB (mean ± STD).

Studies of abnormal cortico‐BG oscillations have focused extensively on the beta band, which can be therapeutically modulated by L‐DOPA therapy (Levy et al. 2002). Recent work suggests that the lower‐frequency bands likely carry BG structure‐specific information, and also can be modulated by dopamine depletion (Gatev et al. 2006; Lemaire et al. 2012; Waldert et al. 2015). In the lower‐frequency bands of the PD patients under investigation, we observed notable differences in the alpha, theta, and delta bands. Using LFP recordings from the same surgery as is depicted in Figures 2, 5A–B represents the mean beta‐band spectral power for striatum, thalamus, and STN. Comparing the individual epoch recordings that occurred within each structure shows that the lower bands are sufficient to distinguish between these three brain areas in this subject (Kruskal–Wallis *H* test, *χ* ^2^(2) = 25.26, *P *=* *3.2 × 10^−6^ striatum vs. thalamus, *P *=* *0.01; striatum vs. STN, *P *<* *0.0001; thalamus vs. STN, *P *=* *0.03). Among the lower‐frequency bands, all three structures are clearly distinguishable within the *θ* and *α* bands. The mean rank of the signal across the trajectory through STN is higher than the signal mean for thalamus or striatum, with a mean striatal signal of 4.63 dB, thalamic of 21.92 dB, and STN of 34.50 dB (mean ± STD).

**Figure 6:**
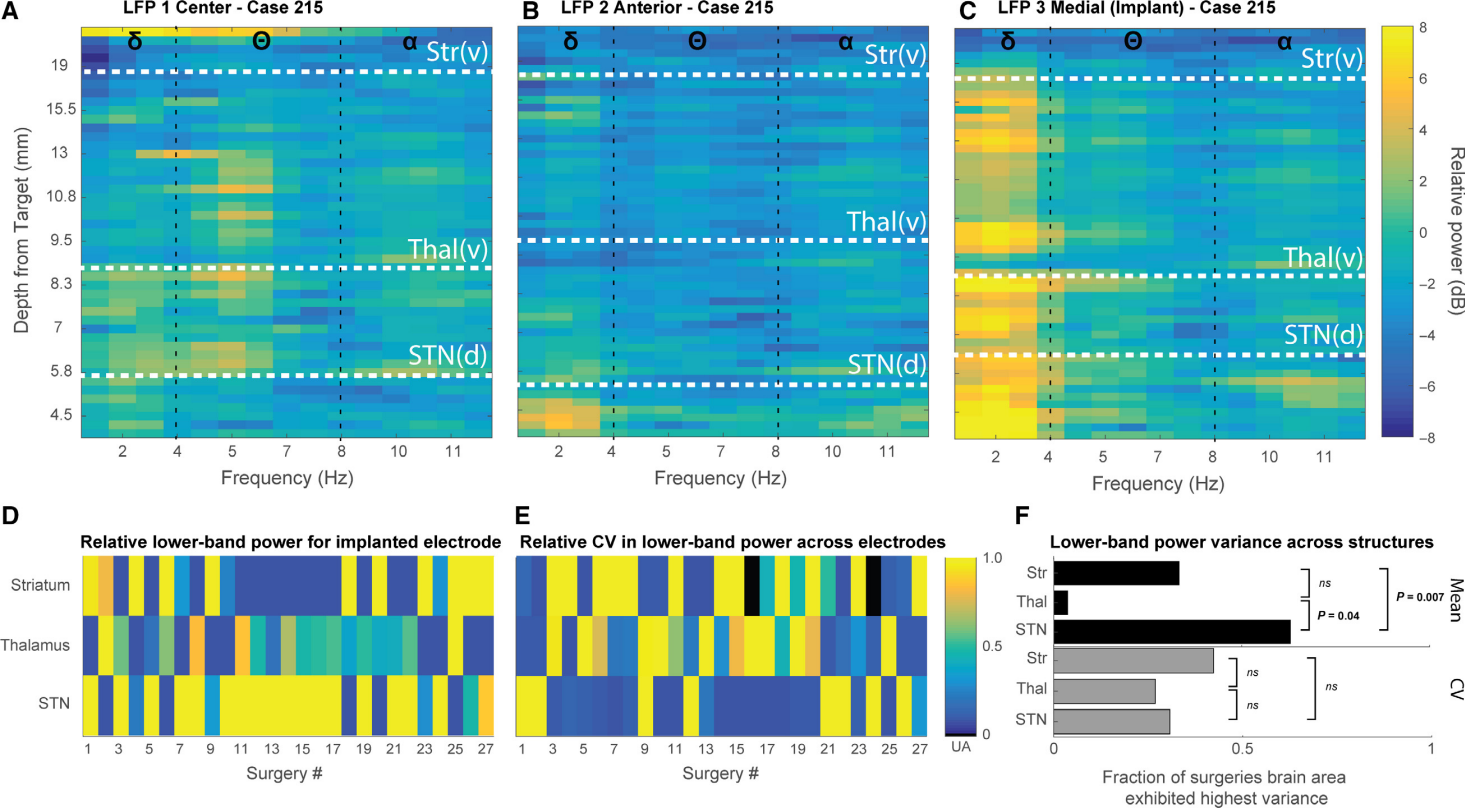
Spectrogram of recording depth over frequency for the delta‐, theta‐, and alpha‐frequency bands. (A–C) Spectrogram of depth over frequency for the delta (1–4 Hz), theta (5–8 Hz), and alpha bands (9–11 Hz) acquired from three simultaneously recorded electrodes during a representative STN deep brain‐stimulating (DBS) surgery. Each graph represents an electrode from a different position within the array: (A) center (derived from direct targeting on preoperative stereotactic imaging), (B) 2 mm anterior to the center, (C) 2 mm medial to the center; trajectory selected for DBS implantation based on single‐unit recordings and intraoperative stimulation mapping. White horizontal lines indicate the borders of the specific brain structures along the trajectory identified using single unit recordings. Frequency bands are delimited by vertical black lines at their borders, and symbol indicated at the top of the graph. (D) Normalized mean low‐frequency (LF; 1–12 Hz) power across structures traversed within the electrode trajectory selected for DBS implantation. Mean LF power for each structure was normalized by the structure exhibited the highest mean power. Each column represents the electrode trajectory selected for DBS implantation for each of 27 surgeries. (E) The coefficient of variation for each structure across all three electrodes for each of the 27 surgeries. (F) Top horizontal bar chart (black bars) summarizes relative mean LF power across implanted electrodes. Statistical analysis using one‐way ANOVA for structure shows that relative mean LF is highest in STN and the thalamus is not different from striatum (*P* < 0.05 for STN vs. striatum and STN vs. thalamus; group means for STN, thalamus, and striatum, respectively: 0.7, 0.34, and 0.42). Bottom horizontal bar chart (gray bars) summarizes CV analysis across all surgeries. For the LF band, across surgeries, no statistical differences were detected.

To further characterize the utility of lower‐band LFP PSD for identifying electrical signatures of BG nuclei, we computed spectrograms of depth versus frequency, focused on the lower bands of interest (Fig. 6A–C). Comparison of the lower‐frequency bands across all trajectories revealed far greater interelectrode and intersubject variation than was found in the beta band (Fig. 6E). In the top row (Fig. 6A–C), in which the plots correspond to a single STN‐DBS implant surgery, the theta band in the center electrode appears to distinguish the thalamus from STN and, in the anterior track (Fig. 6B), the delta band appears to distinguish STN from thalamus. However, in the implanted track (Fig. 6C; medial track), the delta band serves to distinguish STN from the thalamus and striatum. As in Figure 3D–F, we sought to assess population estimates for relative maximal lower‐band activity and identify which structure exhibited the greatest variability in lower‐band activity.

To assess whether increased lower‐band power typified any of the three structures under investigation with respect to the implanted trajectory, we examined the relative power averaged for each structure within the electrode trajectory selected for implantation for all surgeries (Fig. 6D). We observed that in the majority of surgeries, STN evinced the highest lower‐band power across structures, within the implanted track. In a second population analysis, we sought to estimate the relative variability in lower‐band power across structures for all subjects. Figures 6E–F indicate that across subjects, variability appears to be equally distributed across structures and varies more based on surgery (Kruskal–Wallis *H* test, *χ* ^2^(2) = 3.2, *P *=* *0.2). In Figure 6, we found lower‐band activity similar to beta‐band activity is increased in STN, relative to other structures in the implanted track, however, STN appears to exhibit the greatest variability with respect to beta activity.

### Average relative lower‐frequency power increases from striatum to STN

**Figure 7:**
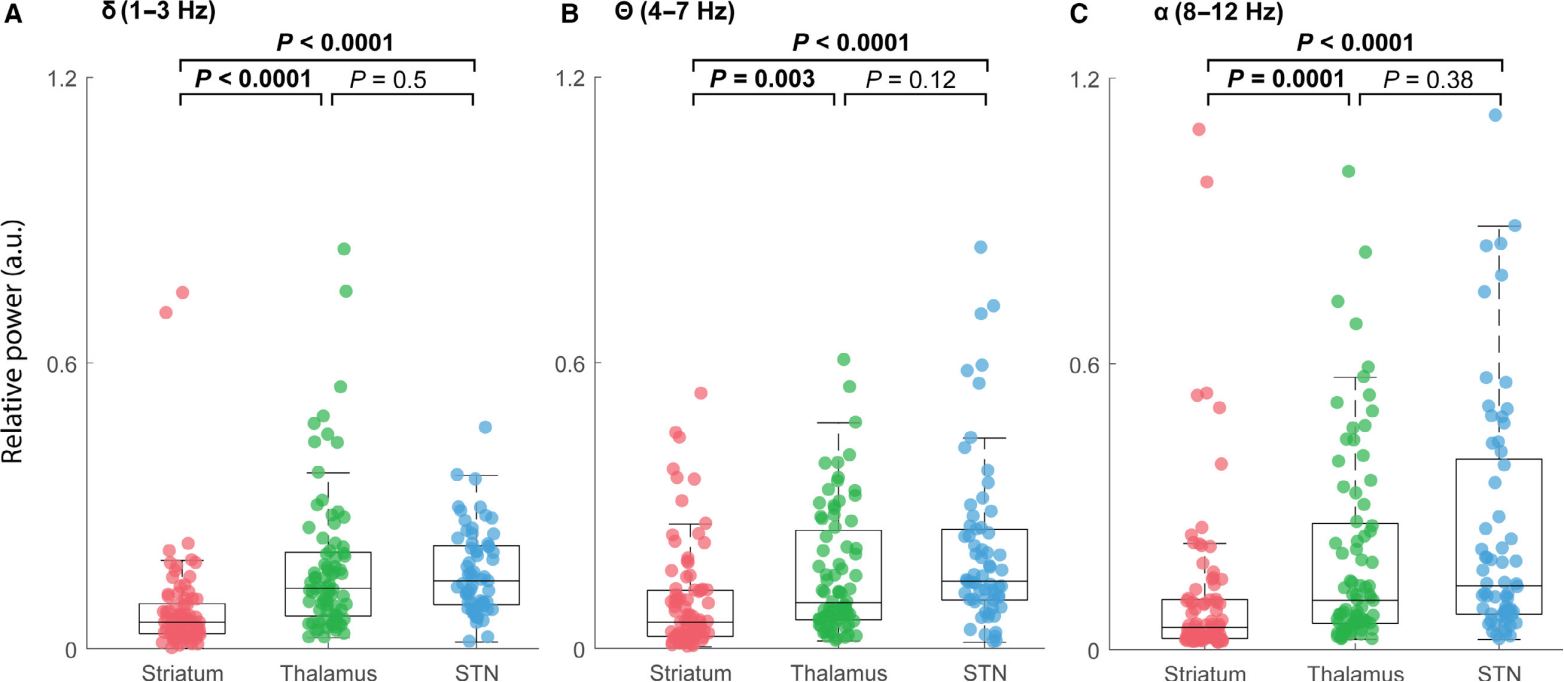
Relative power across structures for all subjects within the lower frequency bands (delta, theta, and alpha). (A) Boxplot of the relative power spectral density for the entire delta‐frequency spectrum (1–3 Hz) comparing striatum, thalamus, and STN for all subjects. Both STN and thalamus exhibited delta band power than striatum, but thalamus was not different from STN. *P*s: striatum versus thalamus, *P *<* *0.0001; striatum versus STN,* P *<* *0.0001; thalamus versus STN,* P *=* *0.55. (B) Similar representation as in (A) with respect to theta frequencies (4–7 Hz). Both STN and thalamus exhibited higher theta power than striatum, but thalamus was not different from STN. *P*s: striatum versus thalamus, *P *=* *0.002; striatum versus STN,* P *<* *0.0001; thalamus versus STN,* P *=* *0.1. (C) Similar representation as in (A) with respect to high beta frequencies (8–12 Hz). Both STN and thalamus exhibited higher‐alpha power than striatum, but thalamus was not different from STN. *P*s: striatum versus thalamus, *P *=* *0.0001; striatum versus STN,* P *<* *0.0001; thalamus versus STN,* P *=* *0.33.

Finally, we again compared the relative band power across structures by normalizing power for each patient based on the average band‐specific magnitude in striatum for each electrode. In Figure 7A, comparing the delta band (1–3 Hz) for all patients across striatum, thalamus, and STN, we found that both STN and thalamus exhibited higher‐delta band power than striatum (Kruskal–Wallis *H* test, *χ* ^2^(2) = 52.95, *P *=* *3.17 × 10^−12^; striatum vs. thalamus, *P *<* *0.0001; striatum vs. STN, *P *<* *0.0001; thalamus vs. STN, *P *=* *0.6; mean ranks: 71.06, 130.91, 142.21, striatum, thalamus, STN, respectively). In Figure 7B and C, we similarly assessed the theta (4–7 Hz) and alpha (8–12) bands, and observed that both STN and thalamus exhibited higher‐delta band power than striatum, but that thalamus and STN were not significantly different. Statistical results for theta: Kruskal–Wallis *H* test, *χ* ^2^(2) = 29.25, *P *=* *4.4 × 10^−7^; striatum versus thalamus, *P *=* *0.002; striatum versus STN, *P *<* *0.0001; thalamus versus STN, *P *=* *0.1; mean ranks: 83.75, 118.68, 141.25, striatum, thalamus, STN, respectively. Statistical results for alpha: Kruskal–Wallis *H* test, *χ* ^2^(2) = 33.04, *P *=* *6.7 × 10^−8^; striatum versus thalamus, *P *=* *0.0001; striatum versus STN, *P *<* *0.0001; thalamus versus STN, *P *=* *0.33; mean ranks: 80.45, 123.72, 139.20, striatum, thalamus, STN, respectively.

## Discussion

In this study, we used intraoperative LFP recordings from PD patients undergoing STN‐DBS implantation to assess whether spectral features across several frequency bands could objectively distinguish between deep brain areas observed during surgical trajectories targeting STN. We found that power spectral analyses of intraoperative LFPs, across several frequency bands (i.e., *δ*,* θ*,* α,* and *β*), could consistently distinguish between BG structures (i.e., striatum, thalamus, and STN) routinely traversed during STN‐DBS implantation surgery. Notably, all structures were differentiable based on high‐beta activity (21–32), but all other bands were only sufficient to differentiate striatum from thalamus and striatum from STN (Figs. 4, 7). Our findings are consistent with the growing body of literature that supports the presence and significance of high‐beta power oscillation in STN (Levy et al. 2000; Brown et al. 2001; Marsden et al. 2001; Brown 2003; Marceglia et al. 2009; Zaidel et al. 2009, 2010), however, our data further demonstrate the novel observation that additional lower‐frequency bands are also higher in STN compared to proximal deep brain structures. These data suggest that aggregate neurophysiological signals, such as LFP, could potentially be used to objectively identify deep brain structures, which would aid in optimizing DBS implantation.

### LFP spectral characteristics throughout CBG circuit in PD

Intraoperative neurophysiological recordings conducted to optimize DBS implantation have revealed prominent enhanced oscillations in beta‐band activity, particularly within STN (Levy et al. 2002; Kühn et al. 2004; Priori et al. 2004; Gatev et al. 2006; Moran et al. 2008). Few studies report on the LFP spectral characteristics from striatal regions, specifically with regard to models of pathophysiological BG. A recent study in normal nonhuman primates demonstrated that beta power recorded from striatum (caudate) was task modulated in the same direction as evidenced in other BG targets; that is, beta power decreased during task execution (Feingold et al. 2015). Moreover, striatal beta power responds similarly to therapeutic high‐frequency stimulation. McCracken and Kiss (2014) observed that striatal LFP recordings in rats show transient reduction in low beta power following entopenduncular nucleus (homolog of human GPi segment) high‐frequency stimulation (McCracken and Kiss 2014). A limitation of our study is that we did not test more than one condition (i.e., resting state) per structure and thus are unable to draw conclusions regarding intrastructural distribution or expression of any particular frequency oscillation.

Investigation of PD‐related pathophysiology of thalamus has largely focused on coherence with cortical activity in the context of tremor generation and less is known about LFP oscillations within thalamus. Sarnthein and Jeanmonod (2007) examined LFP coherence in the pallidal‐recipient thalamic nuclei VA and VLa with scalp EEG in PD patients and found that theta coherence was higher than beta coherence. In contrast, cerebellar‐recipient thalamic nucleus (VIM; ventrointermediate thalamus) evinces the highest coherence with EEG for frequencies in the beta range (8–27 Hz) (Marsden et al. 2000). Our findings demonstrated that thalamic regions traversed during STN mapping exhibit substantial variability in both beta and low‐frequency power. In addition, some areas of thalamus may even exhibit increased beta similar to STN. Our results showed that within beta, both the thalamus and STN expressed greater variability than striatum. In thalamus, this likely reflects transitions between thalamic subcompartments, most notably the dorsal and ventral tier thalamic nuclei (Marsden et al. 2000, 2001; Paradiso et al. 2004; Sarnthein and Jeanmonod 2007). Within STN, observable changes in LFP signal have been noted between the dorsal oscillatory region and the nonoscillatory ventral region (Weinberger et al. 2006; Moran et al. 2008; Zaidel et al. 2009, 2010; Moran and Bar‐Gad 2010). Spatial variation in beta oscillations within different nuclei have been reported to be nonuniform on multiple scales (Moran et al. 2008; Moran and Bar‐Gad 2010; Stein and Bar‐Gad 2013).

Oscillatory activity in STN has been widely studied in the context of advanced PD leading to new perspectives on the pathophysiological CBG circuit (Obeso et al. 2000; Stein and Bar‐Gad 2013; Thompson et al. 2014; Galvan et al. 2015; Guridi and Alegre 2016). Our findings are consistent with previous reports indicating that STN displays the highest beta oscillatory activity (Brown 2003; Doyle et al. 2005; Zaidel et al. 2009, 2010). In addition, our analysis of lower‐frequency bands (*δ*,* θ*, and *α*) revealed differences in power between STN and striatum, as well as thalamus and striatum, but did not distinguish STN from thalamus (Figures 5, 6, 7). Previous studies have shown that some alpha band frequencies show similar pathophysiological increases in CBG structures (Brown and Williams 2005; Rossi et al. 2008). However, our study highlights the role that delta and theta may play in the network dynamics of CBG circuitry. From our investigation of interstructure LFP signatures, only high beta (21–30 Hz) was able to consistently distinguish all structures from one another. We relied on previous designations of low beta (~10–20 Hz) and high beta (~20–30 Hz) (Priori et al. 2004; Avila et al. 2010). Some evidence suggests that there is a functional significance to the difference between high‐ and low‐beta bands and given the arbitrary delimitation, there may be some rationale to refining the functional boundaries of what is currently defined as the beta‐band proper (i.e., 13–30 Hz) (Priori et al. 2002; Williams et al. 2002). For example, higher coherence between STN LFP and EEG in PD patients has been observed for high beta when compared to low beta, suggesting frequency differences in cortical topography of STN‐cortical coupling (Fogelson et al. 2006). These results extend previous findings that examine PD‐CBG intercircuit interactions using LFPs (Zaidel et al. 2010; Alavi et al. 2013). Our findings have implications for both enhancing our understanding of PD pathophysiology and informing the development of improved targeting through acute or chronic LFP analysis of implanted macroelectrodes (Abosch et al. 2012; Galvan et al. 2015; Telkes et al. 2015, 2016).

### Limitations

Our use of LFP analysis to classify deep brain structures is limited by the surgical approach for STN‐DBS. The process in which the LFP data were recorded did not permit correlation or coherence analysis between structures within individual patients, which would have been highly relevant to understanding the extent of the hypersynchrony well known to occur in the PD‐CBG circuit (Weinberger et al. 2006; Huang et al. 2007; Swann et al. 2015). Thus, using parallel recordings collected at the same depth did not allow us to vary the location of the electrode array and simultaneously record from different nuclei. Also, we only examined LFP for a single condition, resting state. Many studies investigating LFP in PD, particularly the beta band, use two or more conditions to uncover underlying oscillatory structure by comparing state changes. It is likely that we could improve our LFP‐based classification of deep brain structure by including an additional condition, such as movement. Finally, we were unable to include SNr in our analysis due to the construction of the recording electrode and surgical procedure. Thus, we were unable to compare our data with previously published reports that examine oscillations in SNr of PD subjects (Alavi et al. 2013).

## Conclusions

In this study, we report on the use of LFP spectra to objectively distinguish between deep brain structures traversed in PD patients undergoing STN‐DBS implantation. We observed that both low‐frequency bands (delta, theta, and alpha) as well as both low and high beta differentiated striatum from thalamus and striatum from STN, however, only high beta separated thalamus from STN. These results indicate that resting‐state LFP signatures from deep brain structures might be used to provide objective classification of electrode location in PD patients.

## Conflict of Interest

The authors declare no conflicts of interests.
